# Lectures on the Morbid Anatomy of the Serous and Mucous Membranes

**Published:** 1841-04

**Authors:** 


### ART. VI.

*Lectures on the Morbid Anatomy of the Serous and Mucous Membranes.* By THOMAS HODGKIN, M.D., &c. Vol. II. Part I. *On the Mucous Membranes.—London,* 1840. 8vo, pp. 541.

IN noticing the first volume of this work in a former Number (vol. IV. p. 31,) we entered at some length into the pathology of the serous membranes, reserving the consideration of a portion of the volume in which some other subjects were treated of until the remaining lectures should come before us. The time which has since elapsed, however, although it has by no means lessened the value of the information contained in these lectures, has yet taken away from its novelty; and as most of our readers have probably become acquainted from various sources with the substance of Dr. Hodgkin's views upon the nature and general characters of malignant disease, we shall at once pass on to the mucous membranes, which form the subject of the volume before us.

The first lecture, the thirteenth of the series, is on the general anatomy of mucous membranes, and professes to give a general view of their structure and analogies. The relation of this tissue to the cutaneous system is pointed out, and the opinions of various authors as to this point brought forward. Bichat's division of the mucous system into gastro-pulmonary and genito-urinary is then adverted to, and a third section, which may be termed the mammary, consisting of that portion which lines the lactiferous tubes and ducts, constituted. This leads to a consideration of the question of the origin of these membranes from the skin, which is shown from the phenomena of progressive development to be untenable. In his observations upon the structure of mucous membranes, we notice that Dr. Hodgkin is not more inclined to admit the globular theory of elementary texture in the case of these membranes than he is in the case of the serous membranes. He adds, however, that neither Mr. Lister nor himself have been able to detect in the mucous membranes that distinctly fibrous texture which belongs to some at least of the serous membranes, and considers their structure to be of a more amorphous character. (p. 14.) In alluding to the varying extent of these membranes, and to the alternate states of distension and contraction to which they are subjected, Dr. Hodgkin takes occasion to remark that many of their movements are effected "by a layer, in most cases double, of contractile fibres, generally considered as muscular, which may be regarded as an appendage to the mucous membrane, stituting a true panniculus carnosus." He infers, however, from the difference found to exist between these fibres and those of true muscle when examined under the microscope, and from a dissimilarity of their chemical composition, that they form a distinct animal tissue. These are not the only motions which the mucous membranes undergo, since by the aid of powerful lenses ciliary movements have been observed on the free surface of several of these membranes, which it is probable exercise an important part in some of the functions performed by them. (p. 15.)

The observations which follow on the pathology of the mucous membranes embrace their congenital or acquired deficiency and excess; partial acquired redundancy forming polypus; varieties in their secretion; the effects of inflammation; and other subjects to which we shall take occasion to refer in the course of our remarks upon the succeeding lectures.

The mucous membranes of the larynx, trachea, and bronchi, with the terminations of the bronchial tubes, form the subjects of lectures xiv. and xv. A good account of the morbid changes observed in the pulmonary mucous membrane generally is given, but we do not find that it contains anything with which the writings and descriptions of preceding authors have not already made us acquainted. Dr. Hodgkin examines first the mucous membrane of the larynx and upper part of the trachea; secondly, that of the lower portion of the trachea, the bronchi, and their ramification; thirdly, the bronchial cells or terminations of the tubes in the pulmonary tissue. The changes connected with a diseased state of the mucous membrane itself, of the larynx, trachea, and bronchial tubes respectively, differ but little if it all, and might have been classed together with advantage as far as regards the descriptive details. For the most part they may be resolved into the effects of inflammation variously modified, the plastic form giving rise to the formation of false membranes, which when seated in the larynx and trachea constitutes croup. The ordinary form, that in which the matter effused on the surface of the membrane does not partake of the characters of coagulable lymph, varies much in degree as well as in extent, and may present simply a congested state of the capillaries or be attended with more or less thickening, softening, abrasion, or ulceration; the secretions at the same time undergoing every gradation of change from simple mucus to purulent or at least puriform matter with or without admixture with the red particles, serum, or other constituents of the blood. The great difference in a practical point of view and in the physiological effects produced arises from the situation of the parts affected. The larynx and trachea occupying the upper portion and entrance of the air-passages when seriously involved must necessarily present symptoms of very threatening aspect. This arises not only from the danger of immediate suffocation from partial or complete closure of the tube, but also from the secondary effects on the brain. In this respect therefore diseases of very different character are found to present similar symptoms, as for instance, inflammatory œdema of the glottis and croup. It is, however, of great importance, as Dr. Hodgkin justly observes, to form the diagnosis between these two affections during life, as the measures which would be applicable in the former case, and often indeed absolutely essential to save the life of the patients would be in the latter if not injurious certainly of little avail. It is the submucous cellular tissue which is the seat of disease in the œdema laryngis, the mucous membrane itself being usually little if at all altered. This form of disease is commonly found to occur in syphilitic patients who have taken cold while under the influence of ptyalism, and we recollect witnessing a case in which a fatal result ensued from the operation of tracheotomy being declined by the surgeon called in consultation, on the presumption of the trachea being implicated in the disease.

In detailing the morbid alterations found in the bronchial tubes, the author alludes to the dilatation of the tubes first pointed out by Laennec. Three forms of this affection are distinguished by Andral: the first, that in which certain of the subdivisions of the bronchi, instead of becoming gradually smaller, nearly equal in size the tubes from which they arise; the second, where there is an absolute dilatation in one or more tubes, forming a kind of *cul-de-sac*, often of considerable size, which has been compared to the finger of a glove; the third, in which there are numerous alternate dilatations and contractions, somewhat resembling, as Laennec states, the appearance of the podded fronds of the *fucus vesiculosus*. In the first form the mucous membrane of the dilated tubes is often of a livid colour, sometimes softer than natural, and occasionally ulcerated: sometimes, as Dr. Hodgkin observes, of extreme tenuity; in other cases, there is sensible thickening, with cartilaginous hardness of the affected tubes. Where the dilated tubes are numerous, the surrounding pulmonary tissue becomes consolidated; and M. Louis has observed that in such cases, when the consolidation is extensive, a dull sound is occasioned on percussion over the affected part. In the second form, which would seem to be more frequent in children, the dilated tubes are more commonly found to contain fetid mucus and pus; whence the affected organ, when cut into, presents the appearance of containing numerous small abscesses. The third form is the most rare, and like the second is also usually found in the lungs of children, the cavities being, in like manner, filled with muco-purulent matter, and presenting similar characters when the lung is cut open. Among the causes assigned are the occurrence of paroxysms of cough, as in hooping cough, and viscidity of the bronchial secretion, leading to its accumulation in certain tubes or portions of tubes. The former, it is thought, is most likely to be concerned in the production of the first form of dilatation; the latter in giving rise to the second and third forms. In reference to the distinguishing of these dilatations, Dr. Hodgkin observes:

"Although there is little danger of mistaking dilated bronchial tubes in the dead subject, when the examination is made in the mode which I have pointed out, by laying them open from the principal branches, yet, when we come upon them by sections into the substance of the lung, they may be mistaken for abscess in the lung, as I have already hinted, or for an excavation produced by the softening and expectoration of a tubercle, to which the resemblance is particularly close, when the dilated tube and the surrounding pulmonary texture have become indurated. If the section be made near to the spot at which several dilated bronchial tubes unite, the appearance which they present is almost precisely that which is seen on the section of a multilocular tuberculous cavity; yet, even in these cases, error may be avoided, by tracing the tubes to a short distance after the section has been made." (p. 62.)

Various forms of bronchitis and catarrh result from the pathological states of the mucous membrane and its secretion. A deficiency in the quantity of the secretion gives rise to that form of bronchial disease, described by Laennec under the term *catarrhe sec.* An increase of the secretion, with more or less change of properties, characterizes the acute mucous catarrh, the chronic mucous catarrh, the pituitous catarrh, and their varieties, of the same author. Dr. Hodgkin believes "that a very general increase of the secretion of the bronchial mucous membrane, sufficient to produce almost universal mucous rattle, and a corresponding difficulty of breathing, imminently threatening or even causing death, may be occasioned by an irritating cause, the direct application of which may be very partial." (p. 64.) The cases to which he refers, however, scarcely admit of explanation upon this principle; and we should be more inclined to consider them as examples of some general affection of the mucous membrane, such as we often witness in cold, damp situations, supervening upon the more local and limited disease, than as directly taking their rise from that source. The nature of the secretion effused into the bronchial tubes, as we have before remarked, embraces every shade, from the natural transparent mucus up to muco-purulent and puriform matter; it is also frequently complicated with the serum of the blood, or the vessels of the membrane may pour out blood itself in large quantities. Like the mucous lining of the larynx and trachea, the bronchial membrane is, in like manner, occasionally the seat of a plastic form of secretion. Dr. Hodgkin mentions a case; and several will be found in some of the older writers, to whom, however, the real nature of the affection was apparently unknown.

In inflammation the membrane is found variously altered, usually vascular and injected, and presenting different shades and degrees of redness; sometimes it is of a bright and deep scarlet, at other times purple brown, or livid. The livid tint is probably most usual in the chronic forms of bronchitis; but in these, especially when there is a free and passive secretion, the membrane is not unfrequently pale throughout. Dr. Hastings states, that in the bronchitis supervening upon measles, he has found the mucous membrane red in patches which approach nearly to the shape of a crescent. He has also observed ulcers in those dying of variola, similar in size to the pustules on the skin. According to the experience of Dr. Hodgkin, ulceration in the bronchial tubes, unless in connexion with tubercles, is of rare occurrence; in which statement he is borne out by Laennec. According to M. Louis and Dr. Stokes, however, ulcers are more frequently met with in the bronchial membrane; and, in many of Dr. Hastings's cases of the chronic form of bronchitis, small ulcers and superficial ulcerations were observed, the membrane being at the same time variously congested and sometimes thickened.

The third portion of the pulmonary mucous membrane, or that lining the air-cells, will require a rather more extended notice. The following method, for which the author acknowledges his obligations to the late Dr. Babington, is recommended as well calculated to show the ultimate ramifications and terminations of the bronchial tubes.

"A collapsed portion of a healthy lung should be taken, having as small an incised snrface as possible; and, on this account, one of the lobes of the lung of an inferior animal answers remarkably well. This portion of lung should then be injected, from the bronchial tube, with the white of egg, in sufficient quantity to distend it, and render its pleural surface smooth. The bronchial tube and the incised surface are then to be secured by ligature, and the whole boiled for a sufficient length of time firmly to coagulate the albumen. By the same process the cellular membrane is so much softened as greatly to facilitate the separation of the structure of the lung without injuring the albumen, which has taken the impression of the cavities into which it was injected. In this way we may discover that nearly all the bronchial ramifications lose their fine tubular form, when they have arrived at a particular degree of minute subdivision; and that, beyond this point, the injected albumen is infiltrated through a spongy texture, so minute that not only its precise form cannot be made out, but its white colour is lost and converted into a gray, from its intermixture with the structure forming the minute cavities in which it is situated." (pp. 79-80.)

Two opinions have been advanced, as our readers are aware, respecting the terminations of the bronchial tubes; one, that there is a free communication between the air-cells and the cellular tissue of the lungs; the other, that the ultimate ramifications of the bronchi are individually unconnected with each other, or with the surrounding pulmonary texture. It would seem from the above method of examination, as well as from others, as Dr. Hodgkin remarks, that there is a communication existing among the air-cells, but that this communication is not indefinitely extended throughout the whole pulmonary tissue.

In relation to the anatomical characters of this, the lining membrane of the air-cells, the question as to the serous or mucous character of the tissue is considered. Unquestionably there are some points of difference between the pulpy villous surface of the better-defined forms of mucous membrane and the attenuated texture of this portion of the bronchial membrane. The transition, however, is gradual, and its appearance analogous to what is observed in the conjunctiva of the eye and in the membrane lining the tympanum, while some of the characters of the serous membrane are wanting. The spongy tissue in which the minute ramifications of the bronchi terminate is regarded as a modification of the cellular membrane.

Passing over various irregularities of development and arrangement, we come to dilatation of the air-cells, or pulmonary emphysema. The causes of this affection, which has of late been so ably illustrated by MM. Louis and Lombard, would seem to be the same as those productive of dilatation of the bronchial tubes. One curious peculiarity attendant upon this disease, to which we are desirous of drawing attention, as having escaped the notice of many acute observers, is that, although there is a strong tendency, as Laennec remarks, in those suffering under this form of pulmonary disease, to organic changes in the structure of the heart, and especially to hypertrophy of the right ventricle, there would seem to be a corresponding diminution in the liability to inflammation of the pulmonary organs. Dr. Hodgkin follows Dr. Addison in regarding all forms of pneumonia, or inflammation of the substance of the lungs, as having their seat on the internal surface of the air-cells. The arguments in favour of this view are, the existence of the rattle, the changes taking place in the expectorated matter when the complaint terminates in resolution, and some phenomena occurring in connexion with pulmonary apoplexy, as it is termed, or the effusion of blood into the air-cells. These characters, it is inferred, necessarily derive their origin from the bronchial membrane and its secretions being implicated—a conclusion which we are by no means disposed to deny; but we cannot see that the fact of this tissue partaking in the morbid action by any means excludes the surrounding cellular texture from being itself the main or primary seat of the inflammatory process.

It is well known that the leading appearances recognized as deriving their origin from inflammation of the pulmonary structure are those designated as engorgement, red hepatization and gray hepatization, and that these are usually considered as indicating different stages of the same process; the engorgement making the first stage, and the red and gray hepatization the successive and more advanced steps of the morbid action, in inducing disorganization of the texture of the lung. Dr. Hodgkin is disposed to take a different view of these appearances, and regards some of them, at least, as being connected with and dependent upon a difference in the process of inflammation itself. We shall endeavour to give a condensed account of his opinions upon this subject, as they involve considerations of some importance as well on practical as on theoretical grounds. It will be recollected that, in treating of the serous membranes, as well as in examining the lesions of the lining membrane of the larynx, trachea, and bronchial tubes, two forms or modes of inflammation have been recognized, in the one of which there is secretion or effusion of fibrinous layers susceptible of organization, and assuming a membranous character, in the other the product chiefly consists of serous or watery fluids occasionally holding minute particles in suspension, which, however, show no disposition to cohere together or become organized. To the first of these the term plastic inflammation is applied, to the second that of non-plastic, or inflammation without plastic effusion. In the plastic form of inflammation of the lungs, the most unequivocal specimens of which are met with in children, the effused lymph is almost invariably tinged with the colouring matter of the blood, the substance of the lung loses its spongy texture and becomes perfectly solid, and, instead of its usual light red or grayish colour, generally assumes a dull but deepish red, very much resembling that of a tolerably healthy liver. Thin slices, carefully washed in water, although they may lose some of their colouring matter, do not allow of the deposit being washed out, and, consequently, do not recover their cellular structure. (p. 94.) Dr. Hodgkin thinks that he is borne out in considering this red hepatization as the product of the plastic form of inflammation by a case in which it was combined with plastic effusion in the bronchial tubes leading to the affected portions of lung, similar to that produced in the trachea in well-marked croup. This alteration is most usually seen in the lobular pneumonia of children, but is sometimes also met with in adults, both in the lobular form, and more diffused through the lung. The author is of opinion that the puckering or contracted appearance, not unfrequently observed on the surface of the lung, is occasionally owing to the loss of substance, contraction, and induration of the cellular tissue, which should follow the subsidence of the inflammation. "On cutting into the substance of the lung," he observes, "to ascertain the state of the interior, on which the superficial appearance depends, we do not always discover either the partially obliterated remains of a tuberculous cavity, or any other indication of the preexistence of tubercular disease, but, on the contrary, a portion of lung remarkable for its induration and solidity, and want of cellular character. It is densest at the centre, whilst its ill-defined circumference passes imperceptibly into the surrounding healthy structure." (p. 97.) We need not tell our readers that Laennec had come to the conclusion that the puckered appearance of the surface of the lung in question depends upon the gradual obliteration of cavities from which tuberculous matter had been removed, and the contraction and drawing together of the pulmonary tissue; and cases are not wanting to bear out his conclusion. The general fact that these peculiar appearances are usually found at some portion of the superior lobes of either lung, that is, in the parts most liable to tubercular deposition, and less frequently the seat of inflammation, is also opposed to the opinion put forward by Dr. Hodgkin; still, we confess, we are more disposed to adopt his view of the case than that of his illustrious predecessor.

The gray or light-coloured consolidation of the lung (gray hepatization, as it is termed) is considered to be the product of the non-plastic form of inflammation, by no means a subsequent stage of the red hepatization, but exhibiting the same pale colour from the very commencement: Dr. Hodgkin is induced to form this opinion, first, because the duration of cases, which, upon inspection, have proved to belong to this form of pneumonia, has been too short to admit of the probability of a transition from the red to the gray having taken place; secondly, that while in the most plastic form of inflammation the bulk of the lung is but little altered, in this form there is often great distension of the pulmonary texture. "On making a section through the affected portion, the incised surface is of a dead soiled white colour, mottled with black pulmonary matter, with a few scattered spots of a reddish colour, generally small and irregular. These red spots are produced by the section of blood-vessels, or by small portions of lung still containing red blood. Slight pressure causes a whitish opaque cream-like fluid to exude from every part of the incised surface." (p. 99.) A thin slice of lung affected with this form of inflammation, if carefully washed, without breaking down the texture of the part, may have the product of the inflammation so completely removed as to show the natural and healthy character of the lung. When this form of pneumonia occupies a considerable extent of the lung it generally proves fatal; when of less extent, the substance of the lung, it is thought, becomes broken down, giving rise to a cavity much resembling those produced by the softening of tuberculous matter, and by its subsequent contraction, sometimes occasioning a puckered appearance on the surface of the lung. It is subsequently stated that every intermediate gradation between these two forms may exist. The more exquisitely-marked states are the most fatal; the intermediate varieties more readily admit of a favorable termination, the deficiency in plasticity, and consequent inferior tendency of the product of inflammation to adhere to the parts producing it, favouring its expectoration, and protecting the air-cells from obliteration on the one hand, while on the other the distension is less and the vitality of the tissue not affected to the same degree as in extreme cases of the non-plastic effusion. (pp. 98-102.)

In addition, however, to engorgement and the two forms of hepatization, with their complications and intermediate states, there have been enumerated among the products of inflammation of the pulmonary texture abscess of the lung, gangrene, pulmonary apoplexy, and œdema of the lung. The first of these is of very rare occurrence, and probably takes place only in the midst of portions of lung in which the air-cells have become obliterated and the texture consolidated by previous inflammation. The pus so formed is, according to Dr. Hodgkin, neither very copious nor very pure; he has never had occasion to see more than a drachm collected in a cavity of this description. The expectoration of purulent matter in considerable quantity, which has sometimes given rise to the opinion of the bursting of an abscess into the bronchial tubes, is now pretty generally admitted to be the result of a communication established between the pleura and the air-tubes in cases of empyema. Other sources of error are the muco-purulent collections found in dilated bronchial tubes; partial and limited empyema of an interlobular fissure, the matter being confined by adhesions towards the edge of the fissure; tuberculous cavities containing puriform secretion, and cavities formed by the breaking down of the lung under the non-plastic form of inflammation. (p. 110.) The remarks on gangrene of the lung present nothing requiring our attention here, unless it be an allusion to its frequent occurrence among the insane, as observed in the lunatic asylum at Ghent. We know not that this observation has been confirmed elsewhere, but we may remark that one of the most marked examples of this state which has come under our notice was in a man affected with delirium tremens.

Pulmonary apoplexy, as it is called, or the effusion of blood into the lungs, though usually resulting from a congested state of the capillary vessels, can scarcely be considered as one of the products of inflammation. A good account is given of the subject, as far as we are acquainted with it; but we may observe that it is one which requires closer investigation than it has hitherto met with to determine the source of the effused blood, and other points of interest connected with it. That the blood rarely proceeds from the rupture of a vessel of any size, is now generally admitted; in which respect a striking analogy is established with hemorrhage from the stomach and intestinal canal generally. To this circumstance we shall have occasion to advert when considering this part of the subject, at present we must be content with quoting the following remark of the author: "I must not omit to state," he observes, "that since the peculiar appearance of the lung, which I have just described as connected with hæmoptysis (small ecchymosed spots scattered throughout the substance of the lungs) first arrested my attention, the examination of one or more recent cases, and the observations of my friend, A. Tweedie, have induced me to feel disposed to unite with him in doubting whether the spotted appearance of the lung has not been the consequence rather than the cause of the hæmoptysis; seeing that it may be brought about by the passage of the blood down the air-tube into the lungs, rather than in the opposite direction." (p. 124.)

In section xvi., Dr. Hodgkin treats of tuberculous deposits in the lungs, prefacing his account by some general remarks on tuberculous matter, its chemical analysis, the variations in form and character which it assumes, its nature and origin, connexion with inflammation, &c. A pretty full account of tubercle, as it appears in the pulmonary organs, is given under the several heads of miliary tubercles; crude tubercles; cavities produced by the expectoration of softened tubercles, a remarkable but frequent form of tuberculous deposit in the lungs which does not appear to have been hitherto distinctly pointed out; tubercles combined with a peculiar form of emphysema of the lung; tubercular infiltration; the stages of crudity, and softening of infiltrated tuberculous matter; tuberculous deposits, succeeding to pulmonary inflammation; and tubercles which do not undergo the process of softening. Some general remarks upon phthisis, and a tabular view of morbid appearances observed in phthisical subjects, drawn up by Mr. T. W. King, from the work or M. Louis, are appended. Several questions of much interest are alluded to in the course of the lecture; but, with the exception of the sections referring to the undescribed form of tuberculous deposit, and to the combination of tubercle with a peculiar form of emphysema, we have not discovered anything with which our readers are not already sufficiently familiar. Under the former of these heads is described the infiltration of portions of the pulmonary texture, varying in size from that of a pea to that of a walnut, or even a small orange, with a grayish translucent effusion, which Dr. Hodgkin regards as an early stage of tuberculous matter. Almost at the circumference of the mass a multitude of small opaque whitish points, similar to those observed in the centre of miliary tubercles passing into a state of crudity, are generally found, and these are so thickly placed together, that, in whatever direction a section is made through the indurated portion, it exhibits a dotted whitish margin, so as almost to suggest the idea of chalcedony set in small pearls. In the translucent gray centre a few small whitish points, less than those which constitute the margin, may frequently be observed. When these become larger and more numerous, and unite, we have a rounded mass of opaque tuberculous matter; and ultimately, when they soften, a tuberculous cavity of the same size. Dr. Hodgkin infers from these appearances an argument in favour of the views of Laennec, and opposed to those of M. Andral, as to the primary character of the gray translucent matter, and the subsequent conversion of the same into the opaque whitish matter. (pp. 164-6.)

In the combination of tubercle with a peculiar form of emphysema, the tuberculous matter is described as occurring in smallish irregular fragments, many of which are accompanied by a cavity containing air in the form of a vesicle, and bearing no resemblance to the ordinary tubercular excavation. Instead of somewhat thickened parietes lined by an imperfect false membrane covered with the *debris* of the tubercle mixed with muco-purulent secretion, the cavities here spoken of have very thin parietes. This vesicular character of the cavities constitutes a peculiar form of emphysema, to be distinguished both from the ordinary enphysema depending upon more or less general dilatation of the air-cells, and from interlobular emphysema of the cellular tissue.

"In the cases now under consideration, although the cavities evidently depend on dilatation of some of the air-cells, yet there does not seem to be necessarily any tendency to dilatation of the neighbouring air-cells. The dilatation appears to be essentially connected with the deposition of tuberculous matter, to which I believe it to be a sequel; since, in the same lung in which these tubercles with vesicles occur, we may find numerous other tubercles without any accompanying vesicle. The tuberculous matter in this cavity appears to be lodged there without any envelope; yet, upon careful examination, in some instances I discovered a very thin and almost imperceptible membrane passing off from the sides of the cavity, and retaining the tuberculous matter in its situation. This membrane appears to throw a little light on the mode in which this complication of emphysema and tubercle is brought about. It would seem that tuberculous matter has been so deposited as to prevent the exit of air, although it allows its ingress: the cells placed under this influence consequently become dilated, and form a somewhat irregular cavity. This distension must of course, in degree, compress the neighbouring pulmonary structure, and bring the tubercular matter to appear as if placed within the cavity; which, indeed, eventually becomes the case, when the delicate membrane before mentioned gives way." (pp. 167-8.)

Before leaving the subject of tubercle of the lung, which, whether in the form of infiltration, or in that of miliary tubercle, is regarded by Dr. Hodgkin as being situated, like the product of inflammation in pneumonia and the effused blood in pulmonary apoplexy, within the air-cells, we must allude to an extraordinary omission of all reference to the views of Dr. Carswell. This is a serious oversight in a work on the mucous membranes, as the effusion of tubercular matter upon the surface of the membrane lining the bronchial tubes is at least as important, and deserves to the full as close an investigation as the varieties of muco-purulent and plastic effusion, or many other subjects treated of in the lectures before us. Perhaps the omission is accounted for by the lecture having been delivered previously to the publication of Dr. Carswell's work, but surely the subject might have been referred to in a note.

The morbid states which form the subject of the seventeenth lecture are for the most part, strictly speaking, foreign to the mucous membranes, though connected with the pulmonary organs. They include the products of disease seated in the cellular structure uniting the lobules, alterations in the vessels and nerves of the lungs, certain adventitious productions, accidental injuries, &c. Some of them would have found a more appropriate place among the lesions of the serous and sub-serous tissues; others might have been considered under the heads of parasitic animals, adventitious structures, and malignant disease, described in the lectures forming a part of the first volume; others again should have been reserved as belonging to the nervous, vascular, and glandular systems. Contenting ourselves therefore with a reference to this lecture, which contains many interesting notices and valuable observations relating to pulmonary diseases, we pass on to the remaining portion of the volume, of which the mucous membrane of the alimentary canal, with its morbid states, forms the subject.

In describing the alterations of structure which are found to occur in this portion of the gastro-pulmonary mucous membrane, Dr. Hodgkin adopts the following divisions: 1, the mucous lining of the mouth; 2, that of the fauces and pharynx; 3, the œsophagus; 4, the stomach; 5, the duodenum; 6, the remainder of the small intestines; 7, the cæcum and colon; 8, the appendix vermiformis; 9, the rectum. The first six of these divisions are considered in lectures xviii. to xxiii.; the last three are reserved for a succeeding portion of the work.

The affections of the mucous membrane of the mouth, fauces, and pharynx, present certain peculiarities, arising partly from exposure, and partly from a difference in anatomical structure. Under this head ptyalism, aphthæ, ulceration and sphacelus, certain diseased states of the tonsil, pharyngeal abscess, &c. are treated of. The account given, however, of some of these states is defective, and exhibits neither the precision nor the comprehensive details which, for the most part, characterize other parts of these lectures. The blue discoloration of the gums in persons affected with lead-poisoning we find no allusion to, and cancrum oris, with its allied forms of disease implicating the mucous membrane and neighbouring tissues, are dismissed with the brief remark, that "in children who have suffered from acute and severe disease, inflammation of the mouth sometimes proceeds to the production of sphacelus of all the textures, not excepting the common integuments. This disorganization proceeds rapidly, produces a dark and almost black colour, and is quickly fatal." (p. 228.) Both these are points deserving of further illustration, the former as likely to prove of material assistance in establishing a correct diagnosis in doubtful cases, the latter as constituting one of the evils occasionally resulting from the confinement and impoverished diet of some of our parochial and criminal establishments. We were desirous of ascertaining whether Dr. Hodgkin, in the course of his extensive experience as a pathologist, had met with any cases of abscess at the back of the pharynx similar to those recently narrated by Dr. Churchill. No example of this kind, however, seems to have come under his notice, though the following case, as far as we can judge from the concise manner in which it is detailed, presents considerable analogy to Dr. Churchill's cases:

"A soldier was admitted into this hospital (Guy's) who had passed a considerable piece of lint up behind the velum palati, where it was completely concealed, being firmly lodged quite at the upper part of the pharynx: this circumstance was not discovered till the body was inspected: his symptoms had been, inflammation about one ear, and *very severe cephalic and thoracic derangement.*

**Extensive phlebitis had been set up, affecting not only the veins of the neck, but the sinuses within the cranium."** (p. 245.)

There is little requiring notice in the observations on the diseased states of the œsophagus. A remarkable case of polypus is quoted from Dr. Monro's work on Morbid Anatomy, and in considering the subject of stricture of the œsophagus some judicious reflections upon the use of the bougie are introduced. The contractile fibrous coat beneath the mucous membrane is regarded by Dr. Hodgkin, notwithstanding its continuity with the muscular expansions of the pharynx, as being of a different character from muscular tissue, and identical in structure with the fibrous coat of the stomach and intestines generally. He observes, "that notwithstanding its fleshy appearance, its fibres, when seen through the microscope, present no trace of that appearance of transverse striæ which is exclusively met with in confessedly muscular fibres; and that it does not, when very thinly extended, moistened, and compressed, exhibit the perfect nacreous lustre which, under these circumstances, he has invariably observed to be distinctly visible in genuine muscle." (p. 260.)

In entering upon the consideration of the morbid changes occurring in the stomach, Dr. Hodgkin very properly commences with an endeavour to ascertain what are the appearances presented by its internal surface in the state of health. Much difference probably exists according to the varying circumstances under which the organ is habitually placed: thus, it has been shown by John Hunter, that an herbivorous animal, the sheep for instance, might be made to eat flesh; and, on the other hand, that the eagle might by education become an exclusively vegetable feeder, such alterations as might have been expected causing a remarkable change in the stomachs of these animals. (p. 265.) Nor can it be doubted that the habitual use of various kinds of diet, as the exclusively or chiefly farinaceous, the vegetable, animal, &c., liquid or solid, mild or stimulant, must exercise an influence on the general state of the mucous membrane and its secretions, without inducing what can properly be termed disease. Very various are the accounts given by different authors who have written upon this subject, of the natural and healthy colour of the membrane. It has been described as being white, grayish white, grayish, reddish, grayish approaching to yellow and red, straw coloured, &c. Billard, in whose opinion Dr. Hodgkin is inclined to place most confidence, states it to be a dead milky white. According to Buisson and Bichat, the colour is of a deep red, and Sabatier and Habicot describe it as of a reddish purple and dull purple. Gavard, Boyer, Sœmmering, Chaussier, and Adelon, make it of various shades between red and grey. Rousseau, who derived his opinion from the examination of the bodies of criminals dying by the hands of the executioner, (by the guillotine we presume,) states that the colour of the gastro-intestinal canal is white, or white faintly tinged with red. Dr. Yelloly states, that in various opportunities which he had of examining the human stomach soon after death, "in such parts of it as were free from vascularity, it had usually a light straw coloured tinge," but gives it as his opinion that "from the analogy of the mucous covering of the mouth and fauces, and of the urethra, it is probable that when circulation is going on in the stomach, its inner surface is of a pale red hue, arising from vessels so minute as to give an uniform colour, without any appearance of distinct vascularity." (Med. Chir. Trans. iv. pp. 393-4.) We are ourselves rather disposed to agree with M. Hippolyte Cloquet, who describes the usual appearance of the membrane as being of a reddish white and mottled (comme marbrée); but we must observe, that this diversity of opinion as to a fact so evident to the senses, could only have arisen from the varying appearances of the membrane presented to the several observers under different circumstances of disease, or from the effects of certain physical agents acting during the last moments of life. The manner of death would appear to exert considerable influence; the presence of aliment recently taken into the stomach causes a decided red tinge throughout the membrane; extremes of cold and heat, according to Beaupré, are also productive of a like effect in the mucous membranes generally, and the stomach has been observed to take a decided tinge from various medicines administered shortly before death. Other variations also, in addition to those of colour, are observed in the mucous membrane of the stomach to the full as important in the due appreciation of its diseased conditions: thus, as Dr. Hodgkin remarks, differences in the thickness and firmness of its substance, and we may add in the varying nature of its surface, may exist between the stomachs of different individuals in perfect health, and causes in themselves trivial, and totally independent of disease, may occasion differences in these respects, which by an inexperienced or incautious observer, might be mistaken for the products of some morbid action. To these variations, however, in equality of surface, in texture and colour, as well as in the appearance of vascular injection not unfrequently presented by the mucous membrane, we shall recur in the course of our observations on various morbid changes taking place in the different portions of the intestinal canal.

The secretions of the stomach are liable to considerable alteration, giving rise to some of the more prominent symptoms of certain forms of gastric disease. With these, however, we need not here delay, but pass on at once to the consideration of the organic changes noticed by Dr. Hodgkin. Preternatural contraction of the stomach is sometimes met with as a consequence of inanition, and especially in connexion with stricture of the œsophagus; but the more usual morbid state in which there is deficiency of the gastric mucous membrane, is when it has been removed by softening and solution, or by ulceration. Dr. Hodgkin alludes to a case related by Dr. Graham of Edinburgh, "in which a very considerable portion of the stomach was supplied by the viscera about that organ; which were so united by adhesion, as to form the parietes of the cavity into which the food was received." (p. 277.) There are many on record of a similar character, though perhaps not equal in extent to the one especially referred to. Of redundancy arising from dilatation of the stomach, there is an interesting collection of instances recorded by Dr. Peebles in the Edinburgh Medical and Surgical Journal for July last, some of which are especially worthy of note as being unconnected with stricture of the pylorus, with which, according to Dr. Hodgkin, this peculiar state of the stomach would seem to be necessarily complicated. There is, however, another form of redundancy, consisting in the preternatural thickness of the membrane to which Dr. Hodgkin assigns the term hypertrophy. This form may be either "the result of an extinct inflammation, or of a preternatural growth from other causes," and is not to be confounded with the thickened pulpy condition often found during states of irritation of the membrane, or in chronic inflammation.

"I should consider," says Dr. Hodgkin, "a portion of the mucous membrane of the stomach of the thickness of about one sixteenth of an inch or upwards as preternaturally thickened. If not actually in a state of inflammation, such thickened mucous membrane, although possibly reddened by the development of its capillaries, might be distinguished from the thickening accompanying present inflammation, by its superior tenacity and firmness, and by the healthy character of the mucus upon its surface. It is not so easy to distinguish those cases of preternatural thickening of the mucous membrane which are the result of inflammation, from those in which it is more probable that inflammation had no part. Yet, on the one hand, we may infer that inflammation has been concerned in producing the thickened state, when we can find traces of ulceration in or near the thickened part, where it is either exceedingly thickened, or inelastic, or excessively lacerable; and, also, when the subjacent cellular membrane is likewise altered in character, having lost its natural laxity, so as to fix the mucous membrane, and interfere with its movements on the subjacent coats. On the other hand, I should feel considerable difficulty in admitting the connexion between inflammation and thickening of the mucous membrane of the stomach, though this were considerable, if no trace of ulceration were discoverable, if the submucous cellular membrane retained its perfectly healthy condition, and if the mucus upon the membrane possessed its healthy and natural character, or, at most, was only rather redundant in quantity." (pp. 278-9.)

Partial hypertrophy of the mucous membrane of the stomach may be observed in that form of disease, designated by Billard and Louis as *mamelonné,* or mamillated. It consists in numerous elevations of a rounded figure, and from two to three lines in diameter, thickly scattered over the surface of the stomach to a greater or less extent, and separated from each other by furrows, in which the membrane is perhaps thinner than natural. Dr. Hodgkin had long been in the habit of noticing this state of the mucous membrane, which he had designated by the term "granular." He regards it as arising from the greater or less development of the natural inequalities existing in the surface of the membrane which are sometimes seen in the healthy stomach when examined early after death. This state is frequently accompanied with ulcerations, from which M. Louis infers that it is a product of inflammation. From this opinion Dr. Hodgkin dissents, although he recognizes a somewhat similar alteration, to which he applies the term fungous as the result of that process.

"In these cases," he observes, "which are comparatively very rare, the elevations bear no resemblance in figure to the natural inequalities before spoken of. They have not the same oblong figure, are rounder, and perhaps more elevated; the mucous membrane is thick, and, containing more blood, is much redder than is natural; the stomach is contracted, and the cellular membrane, as well as the mucous, is much altered." (p. 283.)

This state is mentioned by Billard as occurring in conjunction with disease of the heart. After describing what he terms the inflammatory form of the affection, he proceeds to show that in diseases of the heart, and in those of the great vessels—in a word, in all cases in which the blood is permanently impeded in its course, the cellular tissue becomes infiltrated, œdematous swelling supervenes in almost every part of the body, and the cellular coat of the intestinal canal partakes in the distension, giving to the mucous membrane the œdematous appearance which he had previously described. On the other hand the blood, driven back upon the vena porta and the mesenteric veins, or arrested in the arterial branches, finds its way into the intestinal vessels, and becomes diffused through the mucous membrane, giving rise to redness of that membrane, and increasing yet further the swelling : thence arises a fungous appearance of the inner coat, not of inflammatory origin, and which it is essential not to confound with the results of inflammation. It should be observed that Professor Carswell is inclined to view these elevations as being the follicles of the stomach enlarged, the red spots which are sometimes observed in the centre of the elevations being considered to be the orifices.

In the investigation of those morbid conditions of the mucous membrane which are presumed to indicate the existence of a previous inflammatory state, it is important to bear in mind that some of these are so entirely analogous in their appearance to changes induced in the membrane by other circumstances not connected with disease, as to be frequently with difficulty distinguished from them. Billard, whose work is a valuable contribution to the pathology of the gastro-intestinal mucous membrane, has stated, as we have seen, the usual colour of the internal coat in the state of health to be whitish; but he remarks also, that while the process of digestion is going on, it presents a diffused light rose-red colour. To the accuracy of this observation we can bear decided testimony, as many years ago, and before we had seen the work of this lamented author, we had, from experiments instituted for the purpose, arrived at the same conclusion. Similar appearances, as Dr. Hodgkin states, are produced by the use of alcohol and other stimuli, and the transition from the healthy activity characteristic of the digestive function to that state of irritation bordering upon inflammation, produced by the excessive abuse of some of these stimulants, is so gradual as not to allow of any decided limits to be drawn between healthy action and irritation on the one hand, or between irritation and inflammation on the other. Dr. Hodgkin mentions an appearance which he believes to be essentially characteristic of a recently acute inflammation of the mucous membrane of the stomach, which we neither remember to have met with, nor to have seen described elsewhere. The appearance to which he alludes "is that of an almost infinite number of scattered, small, nearly opaque, whitish spots, which are rather lodged in the mucous membrane itself than situated upon its surface. He has seen it in a case where death arose from prussic acid, and also where arsenic had been taken in a state of solution, but is not prepared to decide whether these spots are depositions of coagulable lymph or not.

Active inflammation of the lining membrane of the stomach is characterized by diffused florid redness of greater or less intensity, with points of still greater intensity, showing a distended state of the capillaries, which may assume either an arborescent appearance, or exhibit numerous small points, striæ, and stars, not unlike in form those of some minute lichens growing on the bark of various trees. From this tension of the capillary system, a degree of softening of the membranes takes place; it becomes easily lacerable, and, during life, in cases of great intensity, blood is sometimes effused, as in other mucous membranes, and in the serous membranes under like circumstances. Sometimes also, as is observed in cases of poisoning with irritant and corrosive poisons, there is exudation of patches of coagulable lymph. This however is a rare occurrence in the stomach, and the acute form of gastritis, except in cases of poisoning, is scarcely ever observed. In speaking of this subject, Dr. Hodgkin takes occasion to allude to the opinions of Broussais, in reference to the connexion of fever with acute inflammation of the stomach, or other portion of the intestinal canal as its cause. We cannot but agree with him in thinking that, however Broussais may have been forestalled in the expression of his leading views by Baglivi, Rega, Prost, Frank, and others, and however erroneous some of his theories may have been, the lasting thanks of the medical profession are yet due to him for calling their attention to this subject, and for much valuable information upon it.

We cannot here follow the author through his description of the ordinary effects of chronic inflammation upon the condition of the membrane. It is sufficient to state that they present little difference from those of the more acute form, except in extent and intensity. It is of more consequence to make our readers acquainted with the marks which he lays down, by which cadaveric changes—the effects of a disordered state of the pulmonary organs, or of diseases of the heart, &c.—may be distinguished from those of inflammation. To assist in the discrimination of a congested state of the mucous membranes, the result of the cases referred to, from the effects of inflammation, attention is drawn to the following points:

"1. The texture of the mucous membrane in a state of injection differs only from the healthy condition in containing a larger quantity of blood, and that for the most part, of a venous hue. It is neither softened nor indurated; except, that the former state may sometimes have been partially induced by the operation of the solvent juices of the stomach, or as a part of the general effect of softening of different textures of the body; amongst which, the mucous membranes of the stomach is the most frequent as well as the most striking seat.

"2. The secretion on the surface of the membrane is not altered in quantity or consistence, except when discolored by transuded blood, when it may receive various degrees of intensity of red, and perhaps some increase of consistence.

"3. The submucous cellular membrane retains its natural texture, and therefore allows the natural mobility of the mucous membrane upon it: it likewise allows of the mucous membrane being torn off in shreds, as in the case of a mucous membrane presenting its most natural appearance.

"4. The vessels communicating with the mucous membrane, but more especially its principal venous branches, are distended and turgid with dark blood: this last appearance is perhaps the most important criterion by which we may be led to distinguish the effects of *congestion* from those of *inflammation*. (p. 295-6.)

In subsequently alluding to the brown and gray discolorations, Dr. Hodgkin advocates the opinion that they are to be regarded either as the effect of a wholly subsided irritation, or of a cadaveric change produced by the action of gases upon the blood in the capillaries. We think there is some reason to doubt the correctness of this view, especially with respect to the brown discoloration, which, although by no means disposed to consider it as being always the product of chronic inflammation, we are yet inclined to look upon as very commonly resulting from some modification of that process.

Ulceration and softening of the mucous membrane of the stomach next engage attention; and under the latter of these subjects we find some important remarks. Dr. Hodgkin is disposed to adhere to the opinion of John Hunter, "that the softening of the stomach is really a cadaveric change, dependent on the structure of the stomach being acted on after death by its own solvent secretion." (p. 306.) We doubt not that, in many reported instances of softening, such may be the case; and we should in all cases be inclined to view the appearance with suspicion when it is conjoined with the presence of crude or partially digested aliment in the stomach. Still, that there are other instances in which the process of softening takes place during life, and is accompanied by certain symptoms which may be taken as characteristic of the affection, we cannot for a moment hesitate to admit; and, indeed, from the subsequent observations of the author, he would himself seem to incline to this opinion—that is, if we understand him aright. He observes, "that the assemblage of symptoms of gastric disturbance, which has been enumerated in connexion with cases of softening of the stomach, is not to be considered as dependent on that derangement of structure, but that the symptoms mark the morbid condition of the stomach in which its vitiated secretion possesses a morbid intensity of solvent power." (p. 309.) The existence, therefore, of the morbid condition is recognized in everything but the production of the softened state of the tissue, it being assumed—for there is no evidence of the fact—that this disorganization does not actually occur until the vitality has ceased.

In connexion with the pathological states of the stomach, there are also examined some forms of malignant disease; the question as to the existence of a follicular apparatus appertaining to the mucous membrane of this part; morbid changes in the sub-mucous tissue, and those seated in the contractile or fibrous coat. Some valuable observations, with cases and experiments upon animals are added, illustrative of the effects of corrosive and irritant poisons upon the inner coat of the stomach, and other parts of the alimentary canal. An important remark, which, from its bearing upon medico-legal enquiries, we must not pass over, is, that where an intense active agent (boiling water and sulphuric acid are especially referred to) has been swallowed or forced into the stomach, it is, as it were, discharged against that part of the internal surface of the stomach which is immediately opposite the opening; and that upon this spot an almost instantaneous change is produced, which is deeper and more intense than that which is afterwards produced in other parts of the mucous membrane, when the agent is diffused over them, lowered in its activity by the mucus, which is rapidly secreted, and which does not merely dilute the noxious agent, but, in some degree, protects the membrane. This spot is that portion of the great curvature situated immediately opposite the œsophagus, whereas, in other cases, the most intense degree of injection is usually met with at the cardiac extremity; when, therefore, this part of the stomach is affected rather than that which is the more common seat of the appearances of inflammation, it may, as the author observes, lead us to the suspicion that some fluid, capable of producing an immediate effect, has been swallowed. (pp. 343-4.)

In the account of the mechanical injuries to which the stomach is liable, we find a case alluded to in which mischief appears to have been done by the incautious use of the stomach-pump. This instrument, it seems, had been employed to remove some deleterious fluid from the stomach of a man in a state of stupor. No relief was experienced, and after the patient's death a few small ecchymosed spots were discovered on the internal surface of the stomach, which corresponded so completely with the end of the instrument as to leave no doubt of their having originated from that source. There is no reason to suppose, it is added, that the fatal result was in any way connected with this injury; but it suggests a useful caution, which those who have occasion to introduce instruments of any description into the stomach will do well to profit by.

The distinction of the three portions of the duodenum, as recognized by Dr. Hodgkin, is well marked by the difference of structure and function by which they are severally characterized, and affords a useful guide in the examination of the lesions of this part of the intestinal tube. Before entering upon the more particular details, certain general deviations from the normal condition, such as the deficiency and excess, dilatation, variations in colour, &c., to which we need not here refer, are pointed out. It should, however, be borne in mind, that, in respect of colour, the mucous membrane of the duodenum, as observed by Billard, certainly differs from that of the stomach, the former being more commonly of an ash colour, not whitish, nor tinged with red, like the latter, excepting during the process of digestion, when, like the stomach, it has more or less of a roseate hue. The first division of the duodenum, the pylori-valvular space of Billard, is that portion which extends from the pylorus to the commencement of the valvulæ conniventes, and is usually about an inch and a half in length. It is in this situation that the solitary glands of Brunner are found in greatest abundance, the whole surface of the membrane being studded with them. The most important morbid alteration discovered here is ulceration, the ulcers being well defined, and of a roundish figure. The ulceration is sometimes connected with tubercular deposition in the sub-mucous tissue, and more frequently still with malignant disease of the neighbouring parts. In the next or middle portion of the duodenum, the valvulæ conniventes are thickly placed, and the orifices of the biliary and pancreatic ducts are here situated: the villi are also very evident in this part. Injection of the mucous membrane is often remarkably apparent, in cases where irritation has existed on the edges of the valvulæ. Many of the French pathologists, belonging to the school of Broussais, give great importance to inflammation of this portion of the intestinal canal, considering many hepatic disorders to take their rise primarily from this source. Dr. Hodgkin, at the same time that he denies the general inference, admits the occasional dependence of jaundice upon an inflamed state of the duodenum. "I have examined," he says, "the bodies of patients who have died in a state of jaundice, in whom no derangement could be detected in the course of the ducts which contained and allowed the passage of bile. In these cases, it seemed highly probable that the impediment had existed at the mouth of the ducts towards the intestine, which is also naturally the narrowest part of the passage. The manifest relief at times afforded by means which act gently on the alimentary canal seems also to favour the idea that the cause of obstruction and the application of the remedy are both to be referred to the duodenal extremity of the ducts." (p. 376.) We may observe that when the difficulty which is not unfrequently found in the discovery of the orifice of the ducts in the healthy state is taken into consideration, it will readily be perceived that a very slight degree of thickening of the membrane, such as must occur in a congested state of the capillaries, may easily be a cause of obstruction, and consequently of jaundice. But however this may be, the practice founded upon the idea that jaundice is dependent upon inflammation of the duodenum will be often found remarkably efficacious. We have often had occasion to see jaundice, in which examination of the region of the liver has afforded no evidence of disease of that organ, give way rapidly to the application of a few leeches to the right of the epigastrium after the use of other remedies had produced little effect; and we cannot but consider that in these cases the relief was probably owing to the removal of an obstruction existing near the opening of the ducts, and dependent upon either an inflamed or congested state of the duodenum. With the exception of such a condition of the lining membrane, this portion of the intestine does not seem to be subject to morbid alterations; we may, however, remark that, although Dr. Hodgkin has himself never heard of strictures existing here, a case is referred to by Dr. Abercrombie (whose work on Diseases of the Abdominal Viscera, by the way, does not seem to have received so much attention from our author as it deserves), in which this lesion was observed, and another is mentioned by Dr. Peebles among the cases of dilatation of the stomach before noticed. The remarks on the biliary ducts, gall-bladder, and pancreatic duct, we are compelled to pass over. The last portion of the duodenum much resembles the jejunum, differing chiefly in its movements, being more restrained, and its valvulæ conniventes and glands being more numerous. The alterations of structure which it presents are either of little importance or resemble those which are found in the jejunum. This portion of the duodenum, therefore, the jejunum, and the upper part of the ileum, have much in common in the general characters of the lesions to which they are respectively subject; these consist in variations in colour, altered secretions, preternatural distension and contraction, inflammatory and congestive injection of the capillaries, thickening, and ulceration: which last, however, is rare in the jejunum, and mostly found in that situation in connexion with tubercular deposition in the submucous tissue.

Intussusception, or the invagination of one portion of the bowel within another, is also a frequent occurrence met with in the small intestines. Of this state, Dr. Hodgkin distinguishes two forms; the first constituting a very serious affection, which is attended with obstruction, vomiting, and all the symptoms of strangulated hernia, is most commonly fatal in its termination, the mucous membrane being found highly vascular and of a livid colour; the other is a cadaveric change, probably, as the author suggests, taking place in *articulo mortis*, and the result of the powerful peristaltic action not unfrequently observed just before death. It is to be distinguished by the absence of all marks of inflammation, the contained portion being a little contracted, and perhaps somewhat paler than the adjoining part. (p. 400.)

In connexion with this part of the intestines, the author enters upon an account of the muciparous glands, and the morbid processes to which they are liable. His descriptions are confined to the solitary glands of Brunner and the aggregate glands of Peyer, the glands of Lieberkuhn and the distinction of those of Brunner into two species, being unknown to him at the time the lecture in which they are noticed was delivered. An account of the admirable researches of Dr. Boehm, to whom we owe a more definite and accurate knowlege of the glandular structures appertaining to the gastro-intestinal mucous membrane, than had previously been acquired, will be found in our Second Number. (Br and For. Med. Rev., vol. I. p. 521.) In the healthy state the solitary glands are small and inconspicuous; sometimes, however, more distinct, especially among children: when diseased they present deviations from the normal structure of much importance; these are described under the heads of increased development or hypertrophy, acute inflammation, and chronic inflammation. The acute inflammation often runs into ulceration, the ulcers being generally small, well-defined, and of a more or less circular figure: many of the instances of perforation of the intestines have arisen from this source. The chronic inflammation of these glands is most commonly observed in connexion with tuberculous deposit, and is also very liable to run into ulceration, being a frequent occurrence in tubercular phthisis. The changes induced by disease in the aggregate glands of Peyer are similar to those which occur in the solitary glands, but differ somewhat in the appearances to which they give rise in consequence of their greater extent and peculiarity of structure. In the acute inflammation of these glands, as in that of the solitary glands, Dr. Hodgkin recognizes two forms: in the first of which he describes the thickness of the patches as being comparatively little increased, while they are reddened by minute and more or less intense injection, in which the surrounding mucous membrane frequently participates. (p. 432.) In the second form there is considerable increase of thickness, the surface becomes uneven, and, as it were, quilted, ulcerated, and of a yellowish or dirty olive colour. In the first form ulceration may also occur, though perhaps more partially than when there is much thickening of the glandular structure. The chronic inflammation and ulceration of the agminated glands is, like the corresponding affections of the glands of Brunner, usually the result of tuberculous deposit.

Dr. Hodgkin has reserved the last portion of the small intestines for separate consideration, on account of the great importance which attaches to the pathological appearances connected with inflammation of this part. The character of these appearances, whether in the primary steps of the inflammatory process or in the ulcerative action which succeeds, presents little if any difference from what has been before noticed as occurring in other parts of the small intestines; but, in consequence of the greater development of the glandular apparatus in the inferior portion of the ileum, and perhaps also from other causes connected with the situation of this part of the bowel, there would seem to be a greater disposition both to inflammation and ulceration here than elsewhere. It is here, therefore, as the author remarks, that the alteration of structure is most frequently met with; it is here, also, that it is met with in its greatest intensity, and when inflammation has affected the glands higher up in the canal, it appears to have been by extension from this part rather than from their having been the primary seat of the derangement. (p. 459.) Our readers are well aware of the views of M. Louis respecting the connexion of fever with a diseased state of the glandular apparatus of this part of the intestinal canal. From his numerous and minute investigations, he has arrived at the conclusion that the acute forms of inflammation and ulceration of these glands are confined to one other disease besides fever, the epidemic cholera, and that the chronic affection is never found, except as connected with the existence of tubercle in the lungs. The application of the numerical test has enabled this faithful and accurate observer to ascertain these facts, the correctness of which, as far as the circumstances of time and situation under which the observations have been carried on are concerned, cannot be questioned. But before we are entitled to extend the generalization to other localities and to other times, it is necessary that researches, conducted with the like patient perseverance and upon an extensive scale, should be entered into, and the results carefully noted and compared. The connexion of the red lenticular patches of the skin with diseases of the glands of Peyer, and consequently with fever, which has been pointed out by the same author, although, in fact, open to observation, has not been so generally recognized as we should have been inclined to anticipate. This eruptive appearance has been noticed by several persons in the fevers of this country, but it has been sought for by others without success; and we find Dr. Hodgkin himself, whose diligence and competence as an observer no one will question, saying, "I have seen—what I imagine all my medical brethren have seen—somewhat of a livid flush on the bodies of fever patients; but I must confess that I had no idea that cutaneous discoloration was either so frequent or so uniform in its character as for it to merit being placed amongst the peculiar symptoms of continued fever; nor am I yet practically acquainted with the appearance alluded to, which I must attribute, in part, to my not having sought for it with sufficient care and perseverance." (p. 461.) With regard to the diseased state of the intestinal glands we find him subsequently making the following remarks: "Although, in my own experience, the derangement of the aggregate glands has certainly been detected in most of the fatal cases of fever which I have examined, I am persuaded that I have examined cases which, during life, had presented the characteristic symptoms of fever, in which the aggregate glands, so far from being severely deranged, have been barely discernible, whilst the cerebral or thoracic derangements have been very considerable. In this assertion I am supported by Dr. Southwood Smith, who has long enjoyed most favorable opportunities for investigating the fevers of the inhabitants of London and its vicinity. Our observations have been made upon the same class of patients, yet our conclusions have been perfectly independent of each other, as I had not until lately consulted his observations." (p. 481.) We feel convinced that such, also, is the general experience of most of those who enjoy the fullest opportunities of becoming acquainted with fever, as it occurs in this country. On the other hand, we believe that a decided predisposition to a diseased state of the mucous surfaces, and especially of the gastro-intestinal mucous membrane, exists in the French capital; and we are inclined to attribute much of the inefficiency of French practice, as well as some of the phenomena presented by disease in that country, to this predisposition.

Dr. Hodgkin, although he admits the frequency of a diseased condition of the glands of Peyer in fever, rejects the idea that it has anything to do with the causation of that disease. In his twenty-third lecture he propounds some opinions of his own regarding the nature of fever, whether it be produced by the influence of some local inflammation or lesion, or exist by itself, independently of such exciting cause. He imagines fever "to depend on the suspension, or, at least, very considerable interruption of that process, by which, during health, the various parts of the system are continually undergoing a change: the old materials being removed, whilst others are substituted in their place;" or, as it is otherwise expressed in the margin, "on the suspension of the universal molecular changes." (p. 490.) We cannot now enter into the examination of these opinions, nor of the arguments by which they are supported; and we are the less disposed to do so, since they are foreign to the general objects of the treatise. We should ourselves have preferred to see the subject of the intestinal mucous membrane completed in this volume, which we conceive might have been readily accomplished without detriment to the arrangement pursued. Dr. Hodgkin's views are, however, always distinguished by good sense, and the practical information which he gives is always sound, and there is no part of the work we have been analyzing which will not well repay an attentive perusal. It is needless to say that we cordially recommend both this and the preceding volume on the serous membranes: they must always rank among the standard records of medical science, and neither the student nor the experienced practitioner ought to be without them.

---

